# Psychological burden, feelings of loneliness and life satisfaction in old age

**DOI:** 10.1192/j.eurpsy.2025.1768

**Published:** 2025-08-26

**Authors:** C. Peglidi, E. Fradelos, F. Malli, A. Zartaloudi

**Affiliations:** 1 University of Thessaly, Larissa; 2 University of West Attica, Athens, Greece

## Abstract

**Introduction:**

As the world population continues to age, understanding the psychological well-being of older people is becoming increasingly vital. Among the various aspects affecting their quality of life, psychological distress, feelings of loneliness and life satisfaction stand out as key dimensions to explore. With age, individuals face a multitude of physical, social and emotional changes that can significantly affect their overall well-being. Consequently, examining the complex relationship between these factors provides valuable insights for promoting healthy ageing and improving the overall quality of life of older people. Psychological distress, often resulting from a range of factors such as chronic health conditions, cognitive decline and social isolation, can manifest itself in various forms, such as anxiety, depression and stress.

**Objectives:**

To investigate the correlation between psychological burden (depression, anxiety, and stress), feeling of loneliness and satisfaction with life among elderly.

**Methods:**

The sample consisted of 148 elderly people over 65 years old. The research instruments used were a) the Depression Anxiety Stress Scale-21, b) the Life Satisfaction Index, c) The UCLA Loneliness Scale, and d) the Athens Insomnia Scale (AIS).

**Results:**

There is a statistically significant association between engagement in domestic activities and a reduction in depressive symptoms. The frequency of children’s visits and the presence of social support networks significantly influence psychological burden. Those who received infrequent or no visits from their children exhibited higher levels of depression. Loneliness was affected by family interactions, and life satisfaction was influenced by gender and education. Participants who had people in their immediate environment helping them with daily needs reported reduced depressive symptoms. Finally, the study revealed statistically significant differences in reported life satisfaction based on participants’ gender and educational level.

**Conclusions:**

These findings emphasize the need for personalized interventions that acknowledge the complex interplay of these factors in shaping the mental health of older adults.

**Disclosure of Interest:**

None Declared

